# Mechanism exploration and prognosis study of Astragali Radix-Spreading hedyotis herb for the treatment of lung adenocarcinoma based on bioinformatics approaches and molecular dynamics simulation

**DOI:** 10.3389/fchem.2023.1128671

**Published:** 2023-03-29

**Authors:** Junfeng Guo, Yuting Zhao, Xuanyu Wu, Ganggang Li, Yuwei Zhang, Yang Song, Quanyu Du

**Affiliations:** ^1^ Hospital of Chengdu University of Traditional Chinese Medicine, Chengdu, China; ^2^ Laboratory of Metabolomics and Drug-Induced Liver Injury, Frontiers Science Center for Disease-related Molecular Network, West China Hospital, Sichuan University, Chengdu, China

**Keywords:** Astragali Radix, spreading hedyotis herb, prognosis, network pharmacology, molecular docking, molecular dynamics simulation

## Abstract

**Background:** Herb pair of Astragali Radix (AR) and Spreading Hedyotis Herb (SH) has been frequently prescribed in clinical for the treatment of lung cancer owing to its favorable efficacy. Yet, the mechanism under the therapeutic effects remained unveiled, which has limited its clinical applications, and new drug development for lung cancer.

**Methods:** The bioactive ingredients of AR and SH were retrieved from the Traditional Chinese Medicine System Pharmacology Database, with the targets of obtained components predicted by Swiss Target Prediction. Genes related to lung adenocarcinoma (LUAD) were acquired from GeneCards, OMIM and CTD databases, with the hub genes of LUAD screened by CTD database. The intersected targets of LUAD and AR-SH were obtained by Venn, with David Database employed to perform Gene Ontology (GO) and Kyoto Encyclopedia of Genes and Genomes (KEGG) enrichment analyses. Survival analysis of the hub genes of LUAD was carried out using TCGA-LUAD dataset. Molecular docking of core proteins and active ingredients was performed by Auto-Dock Vina software, followed by molecular dynamics simulations of protein-ligand complexes with well-docked conformations.

**Results:** 29 active ingredients were screened out with 422 corresponding targets predicted. It is revealed that AR-SH can act on various targets such as EGFR, MAPK1, and KARS by ursolic acid (UA), Astragaloside IV(ASIV), and Isomucronulatol 7,2′-di-O-glucoside (IDOG) to alleviate the symptoms of LUAD. Biological processes involved are protein phosphorylation, negative regulation of apoptotic process, and pathways involved are endocrine resistance, EGFR tyrosine kinase inhibitor resistance, PI3K-Akt, and HIF-1 pathway. Molecular docking analysis indicated that the binding energy of most of the screened active ingredients to proteins encoded by core genes was less than −5.6 kcal/mol, with some active ingredients showing even lower binding energy to EGFR than Gefitinib. Three ligand-receptor complexes including EGFR-UA, MAPK1-ASIV, and KRAS-IDOG were found to bind relatively stable by molecular dynamics simulation, which was consistent with the results of molecule docking.

**Conclusion:** We suggested that the herb pair of AR-SH can act on targets like EGFR, MAPK1 and KRAS by UA, ASIV and IDOG, to play a vital role in the treatment and the enhancement of prognosis of LUAD.

## 1 Introduction

Lung cancer, the major cause of cancer-related mortality around the world, resulted in 1.6 million deaths each year with a poor 5-year survival rate of only 19% (Bray et al., 2018; Siegel et al., 2019). Lung cancer can be categorized into small cell lung cancer (SCLC) (15%) and non-small cell lung cancer (NSCLC) (85%) based on the pathological characteristics and differentiation degree of cancer cells, where the latter is further divided into adenocarcinoma, squamous cell carcinoma, and large cell carcinoma (Sher et al., 2008). Lung adenocarcinoma (LUAD) is one of the common types of lung cancer, accounting for approximately 40% of all lung cancers, which originates from small airway epithelial, type II alveolar cells that secrete mucus and other substances (Noguchi et al., 1995; Zappa and Mousa 2016). Expression of mutated oncogenes in cells can lead to the activation of downstream signaling molecules that drive the abnormal proliferation and differentiation of cells to form tumor cells eventually. Various target agents have been developed that are effective and have low toxicity, but the therapeutic effect of targeted therapy remains unsatisfactory (Eguchi et al., 2008; Yue et al., 2018; Zhong et al., 2021). Although great efforts have been made over the decades, LUAD remains a persistent disease, making it increasingly imperative to search for more effective therapies and drugs for LUAD.

Traditional Chinese Medicine (TCM) pays attention to the enhancement of healthy Qi in patient and individuated therapy for each person in the treatment of lung cancer, which has been widely applied in clinical practice. The advantages of TCM therapy for cancer are extensive, including the improved survival quality of patients (Duflos et al., 2002; Efferth et al., 2007), enhanced physical fitness of patients, alleviation of clinical symptoms, minimum side effects, reduced side effects by radiotherapy, prolonged survival with tumor (Tian and Liu, 2010), and extended survival time (Liao et al., 2017). Modern pharmacological research have demonstrated that TCM and its extracts can act on tumor cells through multiple targets to inhibit the proliferation and migration of tumor cells, playing an essential role in all stages of tumor therapy. TCM has shown potent therapeutic effects to enhance efficacy and reduce toxicity in the complementary treatment of lung cancer, but the underlying molecular mechanisms are too complex and have yet to be revealed.

Through data mining, we discovered that herb pair of AR-SH was most frequently used in the treatment of lung cancer in clinical (Chen et al., 2022). AR, one of the most commonly used tonic herbs in clinical practice, can strengthen the spleen, and enhance the body, where modern pharmacological studies have shown that AR has a wide range of effects including hepatoprotective, diuretic, hypotensive, and immunomodulatory functions (Bedir et al., 2000). Extracts of AR has been widely used as alternative therapies in the treatment of various diseases, including fatigue, anorexia, anemia, fever, allergies, gastric ulcers, and cancer (Astragalus, 2003; Fu et al., 2014). SH is a famous herb with heat-clearing and detoxifying properties, possessing several biological activities, such as neuroprotection (Kim et al., 2001) and antitumor activity (Lee et al., 2011). The anti-tumor effect of SH is generally recognized. SH has been shown to inhibit angiogenesis of tumor (Lin et al., 2011), combat HepG2 cancer cells through inducing apoptosis (Li et al., 2016), effectively kill human colorectal cancer cells (Lin et al., 2015) and breast cancer cells (Liu et al., 2010).

**FIGURE 1:**
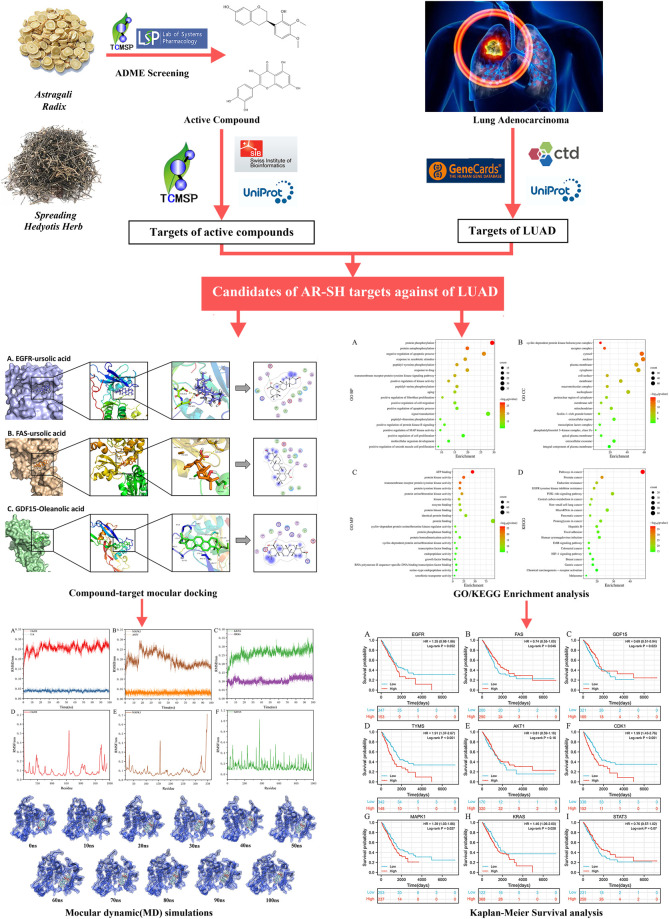
Flow chart of the employed systemic pharmacology strategy.

Network pharmacology is a popular method for predicting the underlying mechanism of herbal medicines. Prognostic analysis is an essential way to evaluate the efficacy of antitumor drugs, and molecular docking (Wang and Zhu 2016), and molecular dynamics simulations (De Vivo et al., 2016) can be used to validate and complement the network pharmacological results. In this study, a systemic pharmacology strategy (Figure 1) integrating network pharmacology, molecular docking and molecular dynamics simulations, was employed to explore the active components of AR-SH, and their corresponding targets and signaling pathways in the treatment of LUAD, with prognostic analysis used to examine key targets of AR-SH so as to provide scientific evidence for the complementary therapeutic effect of AR-SH in LUAD.

## 2 Materials and methods

### 2.1 Collection of active compounds and targets prediction of AR-SH

The active compounds of AR-SH were searched using the Traditional Chinese Medicine Systems Pharmacology (TCMSP, https://old.tcmsp-e.com/tcmsp.php) with the criteria set as oral-bioavailability (OB) ≥ 30% and drug-likeness (DL) ≥ 0.18. In addition, bioactive ingredients of AR-SH were supplemented from relevant literature. Pubchem database (https://pubchem.ncbi.nlm.nih.gov/) were employed to acquire the 2D structure of the ingredients, which were further uploaded to Swiss Target Prediction (http://www.swisstargetprediction.ch/) for target prediction with screening standard as Probability≥0.1. We calculated the similarity matrix of the molecules by Morgan Fingerprint in the RDKit toolkit, and the similarity was evaluated using the Tanimoto score (Hert et al., 2004; Rogers and Hahn 2010).

### 2.2 Acquisition of LUAD genes and screening of hub genes

LUAD related genes were obtained using the keyword “lung adenocarcinoma” in various databases, including GeneCard (https://www.genecards.org/), Online Mendelian Inheritance in Man (OMIM, https://omim.org/) and Comparative Toxicogenomics Database (CTD, http://ctdbase.org/). The genes retrieved from the databases were integrated and de-duplicated, and the protein names were normalized using the Uniprot (https://www.uniprot.org/) database. Through the CTD database, the common targets with the highest “Inference Score” and “References” were selected as hub genes of LUAD.

### 2.3 Gene ontology (GO) and kyoto encyclopedia of genes and genomes (KEGG) pathway enrichment analyses

The screened targets of AR-SH and LUAD genes were imported into the Venny2.1 online platform to capture the common targets of active compounds and LUAD. GO enrichment analysis of the intersected targets was performed in terms of the biological process (BP), cellular component (CC), and molecular function (MF) based on the David database. KEGG was selected for target pathway annotation analysis with P set less than 0.05, and the top 20 KEGG signal pathways were ranked according to the results in descending order of enrichment value.

### 2.4 Survival analysis of hub genes of LUAD

Gene expression data and survival information obtained from the Cancer Genome Atlas (TCGA) database were assessed by Kaplan-Meier survival analysis, and a log-rank test was performed using the survival package version 2.44-1.1 in R software.

### 2.5 The molecular docking of active compounds of AR-SH with core proteins of LUAD.

The 2D structure of the screened ingredients were downloaded from the PubChem database, which were imported into Chem3D software to draw the 3D structure of the compounds and optimize the energy of the ligand structure. The processed structure was saved in PDB format, and AutoDockTools-1.5.6 software was then applied to add charge and display rotatable keys, with the final structure saved in PDBQT format.

Next, the protein crystal structures encoded by hub genes were obtained from the PDB database (https://www.rcsb.org/), which were imported into PyMOL software to remove solvent and ligand. AutoDockTools-1.5.6 software was then employed to add hydrogen atoms, and the structure was saved in PDBQT format, with their active pockets searched. Molecular docking was performed by adjusting the X-Y-Z coordinates and grid size of the protein and optimizing the position of the protein structure binding sites. Processed active compound and the protein were docked for ten times by AutoDock Vina with the minimum binding energy of each docking taken as the final result. Docking results of the clinically used epidermal growth factor receptor-tyrosine kinase inhibitor (EGFR-TKI) Gefitinib with the core proteins were compared with those of the screened compounds with the core proteins. Docked ligand-protein complexes with lower docking binding affinity and research value for each protein were selected for further detailed demonstration.

### 2.6 Molecular dynamic (MD) simulation

The conformations of core protein-ligand complexes with lower docking binding affinity and research significance in the molecular docking results were further analyzed by MD simulations. MD simulation was carried out using GROMACS (version 2021-2). Protein topology file was generated using the AMBER99SB-ILDN force field, whereas ligand topology file was generated by ACPYPE script using the AMBER14SB force field. MD simulation was carried out in a dodecahedral box filled with TIP3 water molecules, and periodic bounding conditions were applied. The system was neutralized with NaCl counter ions. Energy minimization was achieved using the steepest descent algorithm, with cutoff of 1.4 nm for Coulomb interactions and Van der Waals interactions.

Before the simulation, each system was equilibrated for 100 ps at 310 K for NVT (constant atomic number, volume, and temperature) using a V-rescale thermostat (Bussi et al., 2007) and for 100 ps at 1.0 bar for NPT (constant atomic number, pressure, and temperature) *via* a Parrinello-Rahman barometer. The protein backbone was inhibited, while the solvent and countercharge ions were allowed to move during the equilibrium phase. The LINCS algorithm was used for all binding constraints. The particle-mesh Ewald (PME) method was used for long-range electrostatic processing. During the simulation, the positional constraints were removed. Finally, simulations were performed for 100 ns for each system under periodic boundary conditions at 310 K temperature and 1.0 bar pressure, and snapshots of the trajectories were taken every 10 ns.

### 2.7 Free binding energy calculations

The calculation of the free binding energy of protein-ligand complexes is an important way to verify the strength of intermolecular interactions, providing insight into the relative importance of various chemical energies that contribute to the overall stability. The molecular mechanics Poisson-Boltzmann surface area (MM-PBSA) method is a simple technique for quantifying the binding free energy of a ligand docked to an acceptor (Miller et al., 2012). The g_mmpbsa (Miller et al., 2012) tool was used to calculate the binding affinity of simulated protein-ligand complexes.

In general, Formula 1 can be used to calculate the free binding energy of a protein to a ligand in a solvent (Kollman et al., 2000):

$$\Delta G_{bind}=G_{complex}-\left(G_{protein}+G_{ligand}\right)$$ (1)

$G_{protein}$ and $G_{ligand}$ denote the total free energy of the isolated protein and ligand in the solvent, respectively, and $G_{complex}$ represents the total free energy of the protein-ligand complex. In addition, the free energy of each entity can be obtained using Formula 2:

$$G_{x}=\left(E_{MM}\right)-TS+\left(G_{solvation}\right)$$ (2)

X denotes protein or ligand or protein-ligand complex. $\left(E_{MM}\right)$ represents the average molecular mechanical potential energy in vacuum. $\left(G_{solvation}\right)$ denotes the solvation free energy (Kollman et al., 2000; Kumari et al., 2014). TS represents the entropic contribution of the free energy in vacuum, where T and S denote temperature and entropy, respectively. The TS term is the conformational entropy term associated with complex, and isolated protein is calculated in the vacuum environment. Instead of considering absolute binding free energy, we focused on the contribution of individual residues of protein and ligands to the individual components of $E_{MM}$ and $G_{solvation}$ terms. The change in entropy term was neglected owing to that it does not affect the relative binding energy of ligands.

The molecular mechanics potential energy E_MM_ is the vacuum potential energy and includes both bonded and non-bonded interactions. It is calculated using molecular mechanics (MM) force field parameters, as in Formula 3

$$E_{MM}=E_{bonded}+E_{nonbonded}=E_{bonded}+\left(E_{vdW}+E_{elec}\right)$$ (3)

The value of $E_{bonded}$ energy can be taken as zero under the assumption that the bound and unbound forms of protein and ligand conformations in the single trajectory method are similar (Homeyer and Gohlke 2012). Non-bonded interactions ( $E_{nonbonded}$ ) include electrostatic ( $E_{elec}$ ) and van der Waals ( $E_{vdW}$ ) interactions.

The free energy of dissolution is the energy required to transfer the solute from the vacuum to the solvent. In the MM-PBSA method, the free energy of dissolution is calculated using the following solvent model, as in Formula 4:

$$G_{solvation}=G_{PB}+G_{SA}$$ (4)

$G_{PB}$ and $G_{SA}$ denote the electrostatic and non-electrostatic contributions to the free energy of dissolution, respectively. The electrostatic term G_polar_ was calculated by solving the Poisson-Boltzmann (PB) equation (Wang et al., 2004), and the G_SA_ term was calculated using the solvent accessible surface area (SASA). We also performed studies related to the energy decomposition of each residue, which help to estimate the MM-PBSA binding energy of the ligand in the protein-ligand complex.

## 3 Results

### 3.1 The active compounds and targets of AR-SH

The active compounds and targets of AR-SH were obtained by searching the TCMSP platform and the Swiss Target Prediction database, respectively. Among them, 29 active compounds of AR were retrieved with 361 targets predicted, and 2 compounds supplemented from literature were Astragalus polysaccharide (Bamodu et al., 2019) and AstragalosideIV (Zhang et al., 2018; Chen et al., 2021). 7 active compounds of SH were retrieved, with 227 targets predicted, and 3 compounds supplemented from literature included ursolic acid, Mairin and oleanolic acid (Liang et al., 2022), as shown in Table 1. 21 of the 29 obtained active compounds were found to possess a diversity index less than 0.8 and an average score of 0.178, which indicated a favorable diversity, as shown in Supplementary Table S1.

**TABLE 1:** Active components of AR-SH.

Num	PubChem CID	Molecule name	Source	MW	OB (%)	DL
1	10380176	(R)-Isomucronulatol	AR	302.35	67.67	0.26
2	15976101	(24S)-24-Propylcholesta-5-ene-3beta-ol	AR	428.82	36.23	0.78
3	11869658	3-Epioleanolic acid	SH	456.78	32.03	0.76
4	14077830	Astrapterocarpan	AR	300.33	64.26	0.42
5	5316760	1,7-Dihydroxy-3,9-dimethoxy pterocarpene	AR	314.31	39.05	0.48
6	2782115	2-(Chloromethyl)-4-(4-nitrophenyl)-1,3-thiazole	AR	254.69	\	\
7	162906151	2,3-dimethoxy-6-methyanthraquinone	SH	282.31	34.86	0.26
8	10514946	2-methyl-3-methoxyanthraquinone	SH	252.28	37.83	0.21
9	15689655	3,9-di-O-methylnissolin	AR	314.36	53.74	0.48
10	162842488	5′-hydroxyiso-muronulatol-2′,5′-di-O-glucoside	AR	642.67	41.72	0.69
11	15689652	7-O-methylisomucronulatol	AR	316.38	74.69	0.3
12	101679160	9,10-dimethoxypterocarpan-3-O-β-D-glucoside	AR	462.49	36.74	0.92
13	222284	beta-sitosterol	SH	414.79	36.91	0.75
14	108213	Bifendate	AR	418.38	31.1	0.67
15	5280448	Calycosin	AR	284.28	47.75	0.24
16	6037	FA	AR	441.45	68.96	0.71
17	5280378	formononetin	AR	268.28	69.67	0.21
18	73299	hederagenin	AR	414.79	36.91	0.75
19	15689653	Isomucronulatol 7,2′-di-O-glucoside	AR	626.67	49.28	0.62
20	5281654	isorhamnetin	AR	316.28	49.6	0.31
21	5318869	Jaranol	AR	314.31	50.83	0.29
22	5280863	kaempferol	AR	286.25	41.88	0.24
23	64971	Mairin	AR	456.78	55.38	0.78
			SH			
24	10494	Oleanolic acid	SH	456.78	29.02	0.76
25	5281330	Poriferasterol	SH	412.77	43.83	0.76
26	5280343	quercetin	AR	302.25	46.43	0.28
			SH			
27	5280794	Stigmasterol	SH	412.77	43.83	0.76
28	64945	ursolic acid	SH	456.78	16.77	0.75
29	122130319	AstragalosideIV	AR	785.09	17.74	0.15

### 3.2 Acquisition of LUAD-related genes and screening of hub genes of LUAD

**FIGURE 2:**
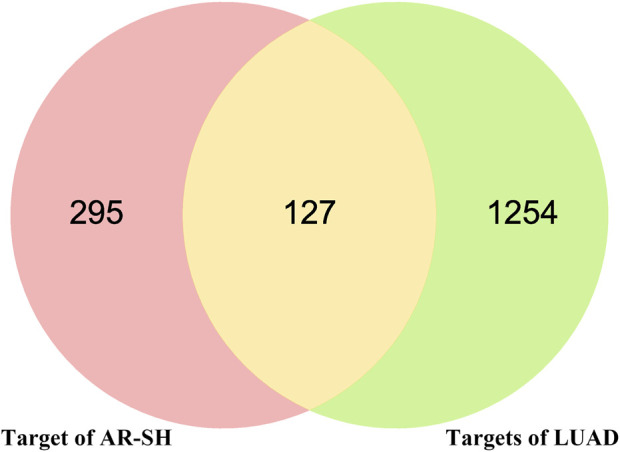
Venn diagram of AR-SH and LUAD intersected targets.

Using Gene Cards, OMIM and CTD databases, 1091, 227 and 157 LUAD-related genes were acquired respectively, with a total 1381 LUAD-related genes obtained after de-duplication. The 422 active compounds targets and 1381 LUAD genes were analyzed by Venn, and 127 common targets were obtained, which maybe the potential targets of AR-SH for LUAD treatment, as shown in Figure 2.

Based on CTD database, 10 hub genes were selected as docking targets for the next simulation experiments according to their “Inference Score” and “References” scores as well as lung adenocarcinoma-related research hotspots. As shown in the Table 2, the proteins coded by the 10 hub genes are Epidermal growth factor receptor (EGFR), Fas cell surface death receptor (FAS), Growth differentiation factor 15 (GDF15), Thymidylate synthetase (TYMS), AKT serine/threonine kinase 1 (AKT1), Cyclin dependent kinase 1 (CDK1)), Mitogen-activated protein kinase 1 (MAPK1), KRAS proto-oncogene, GTPase (KRAS), Signal transducer and activator of transcription 3 (STAT3), and Matrix metalloproteinase-9 (MMP9).

**TABLE 2:** The inference score and reference score of the hub genes.

Gene symbol	Inference score	References
EGFR	41.93	38
FAS	39.02	23
GDF15	35.62	22
TYMS	35.01	24
AKT1	34.17	34
CDK1	33.91	26
MAPK1	32.37	39
KRAS	32.07	33
STAT3	30.93	29
MMP9	28.3	29

### 3.3 GO and KEGG pathway enrichment analysis

GO is a bioinformatics analysis tool that defines the input genes by describing the function of the gene and the relationship between the enriched terms. GO functional analysis divides the gene functions into three parts: cellular component (CC), molecular function (MF), and biological process (BP), among which, BP can best reflect changes in biological function within the body.

**FIGURE 3:**
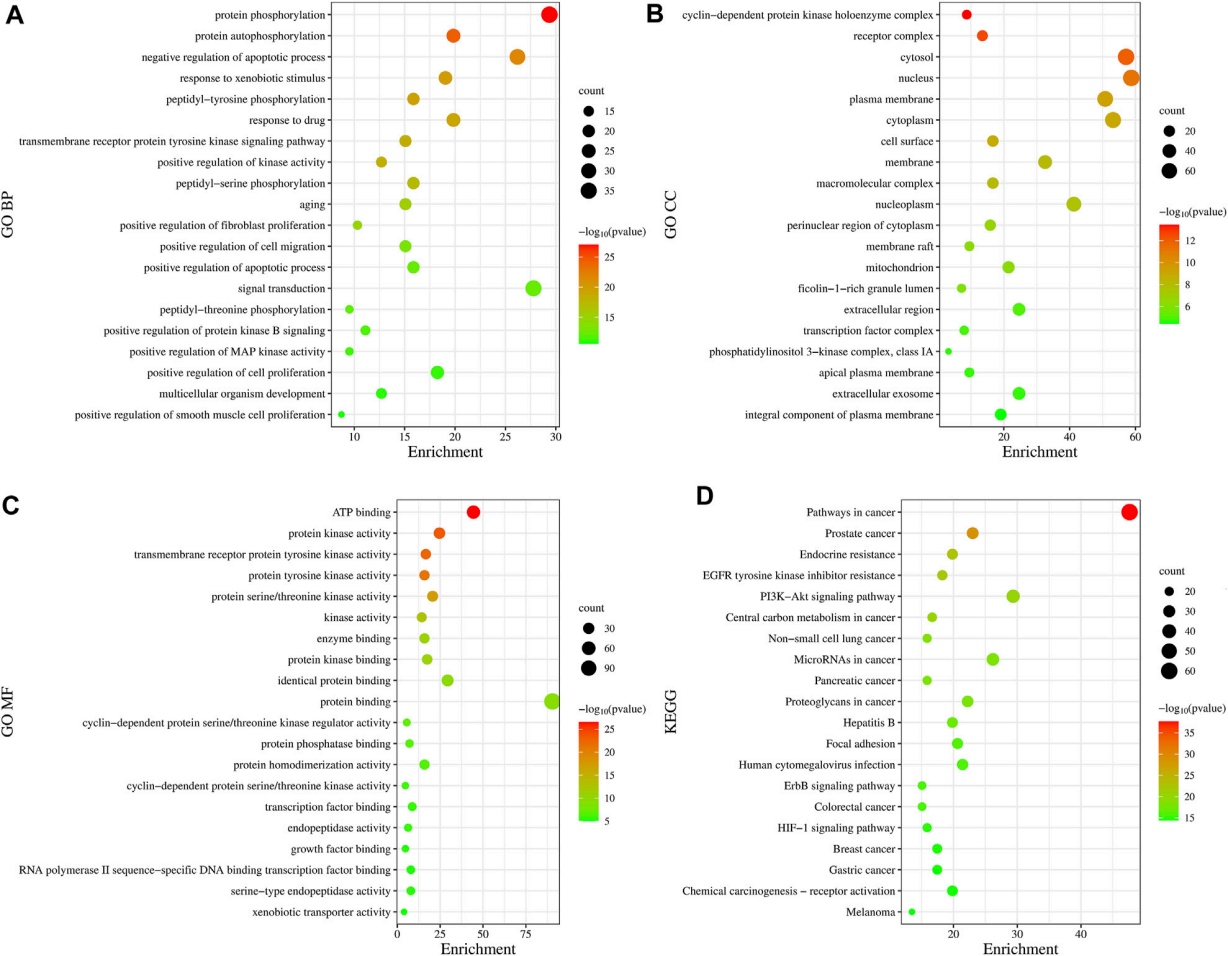
Gene Ontology functional and Kyoto Encyclopedia of Genes and Genomes pathway enrichment analyses. **(A)** Biological Processes. **(B)** Cellular Component. **(C)** Molecular Function. **(D)** KEGG analysis. The size of the dots represents the number of genes; the larger is the dot, the higher is the number of genes in the corresponding process. *p* values indicate the importance of enrichment; the lower is the *p* values, the redder is the color of the graph.

In total, 775 GO entries of the GO functional enrichment analysis were obtained from DAVID database, including 556 entries in BP, 89 entries in CC, and 130 entries related to MF. Figure 3A shows that the potential targets were mainly enriched in BP such as protein phosphorylation, negative regulation of apoptotic process, response to xenobiotic stimulus, peptide-tyrosine phosphorylation and response to drugs. The involved terms of CC shown in Figure 3B are cyclin-dependent protein kinase holoenzyme complex, receptor complex, cytoplasm and plasma membrane accounted for a significant proportion. As for MF, ATP binding, protein kinase activation, transmembrane receptor protein tyrosine kinase activity, protein tyrosine kinase activity and protein serine/threonine kinase activity were ranked in the top, as shown in Figure 3C. Using KEGG pathway enrichment analysis, 147 pathways were screened out based on the threshold of *p* < 0.05. As shown in Figure 3D, the pathways with the highest significance, involve a variety of cancer pathways, including non-small cell lung cancer, prostate cancer, pancreatic cancer, colorectal cancer, etc. Cancer-related cell alterations, including endocrine resistance, EGFR tyrosine kinase inhibitor resistance, central carbon metabolism and proteoglycans in cancer are also enriched significantly. Signaling pathways including PI3K-Akt and HIF-1 also are shown to be important.

### 3.4 Survival analysis of important targets

**FIGURE 4:**
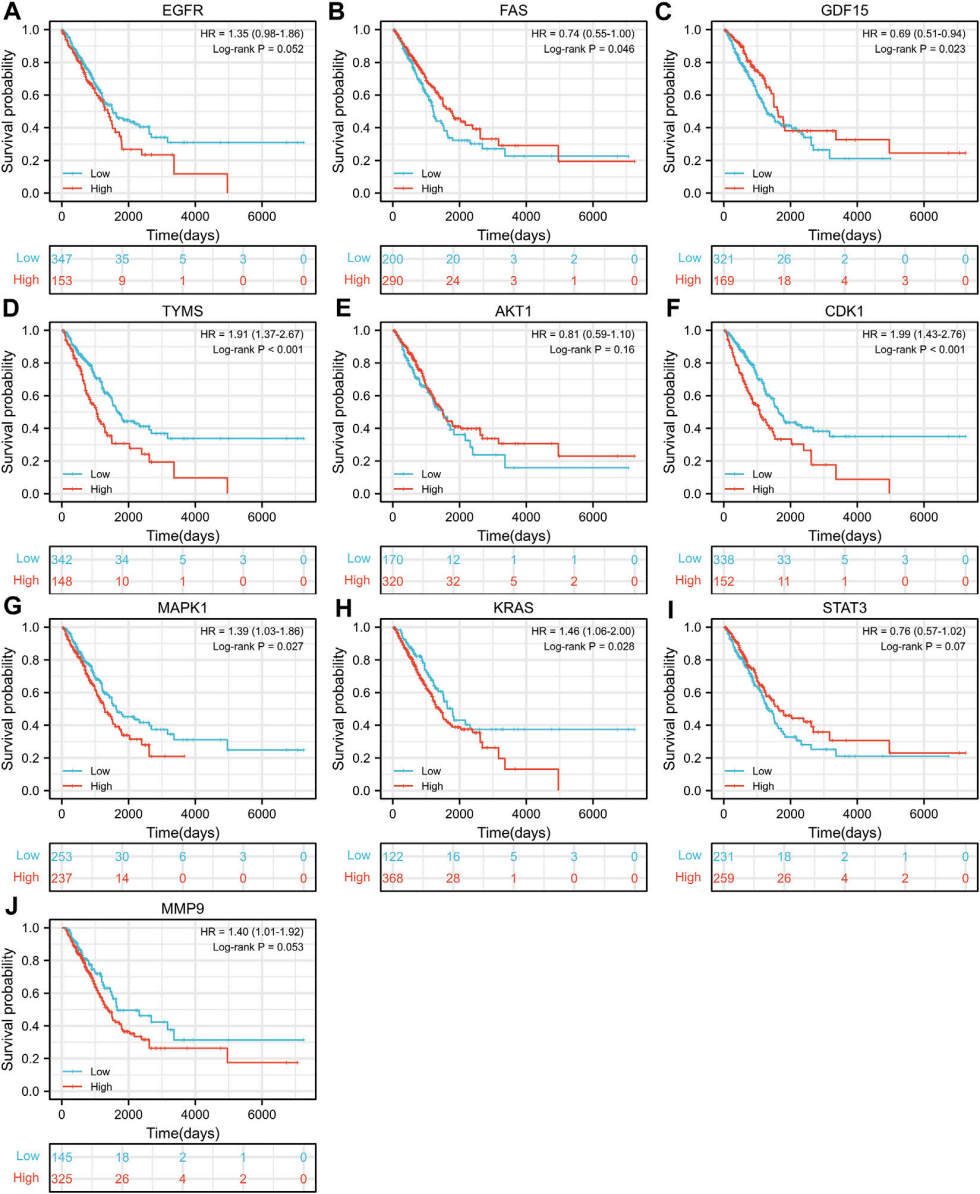
Kaplan-Meier Survival analysis of the correlation between expression of important target genes **(A–J)** and prognosis of LUAD in TCGA database.

TCGA-LUAD dataset consisting of 526 LUAD samples and 59 normal samples were obtained from the TCGA database. We divided the LUAD samples into high and low expression groups according to the expression levels of the ten hub genes, and further investigated the correlation between the expression of the ten hub genes and the prognosis of LUAD patients by Kaplan-Meier survival analysis. As shown in Figure 4, the expression of FAS (*p* = 0.046), GDF15 (*p* = 0.023), TYMS (*p* < 0.001), CDK1 (*p* < 0.001), MAPK1 (*p* = 0.027) and KRAS (*p* = 0.028) showed significant correlation with prognosis. The survival analysis revealed that the correlation between the expression of other hub genes and the survival is not statistically significant. However, EGFR has been proven to be a determinant driving lung adenocarcinoma growth and treatment response *in vivo* (Foggetti et al., 2021), thus subsequent analysis will also be performed for EGFR.

### 3.5 Molecular docking

**FIGURE 5:**
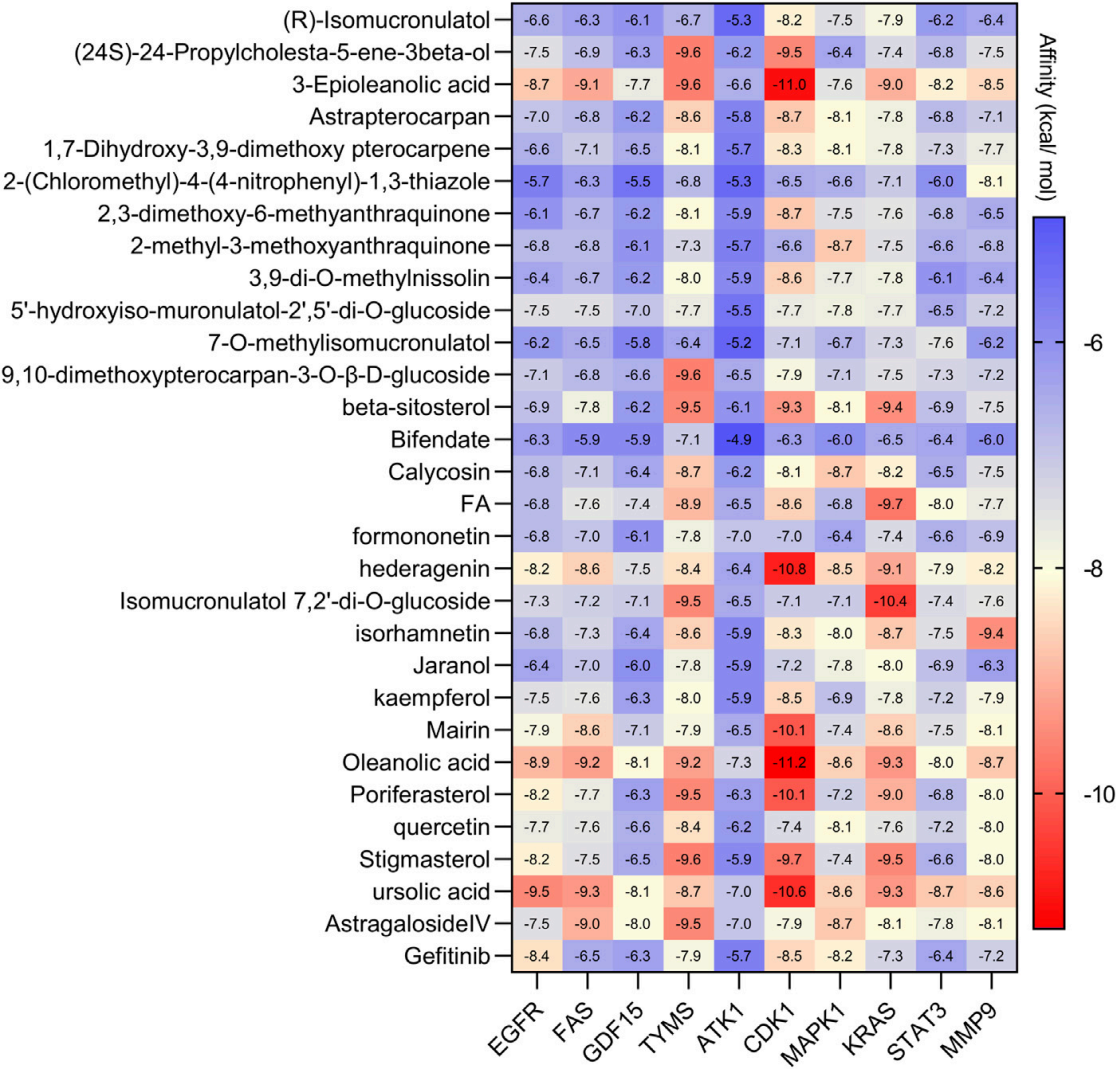
Docking results of active ingredients of AR-SH with core proteins.

**FIGURE 6:**
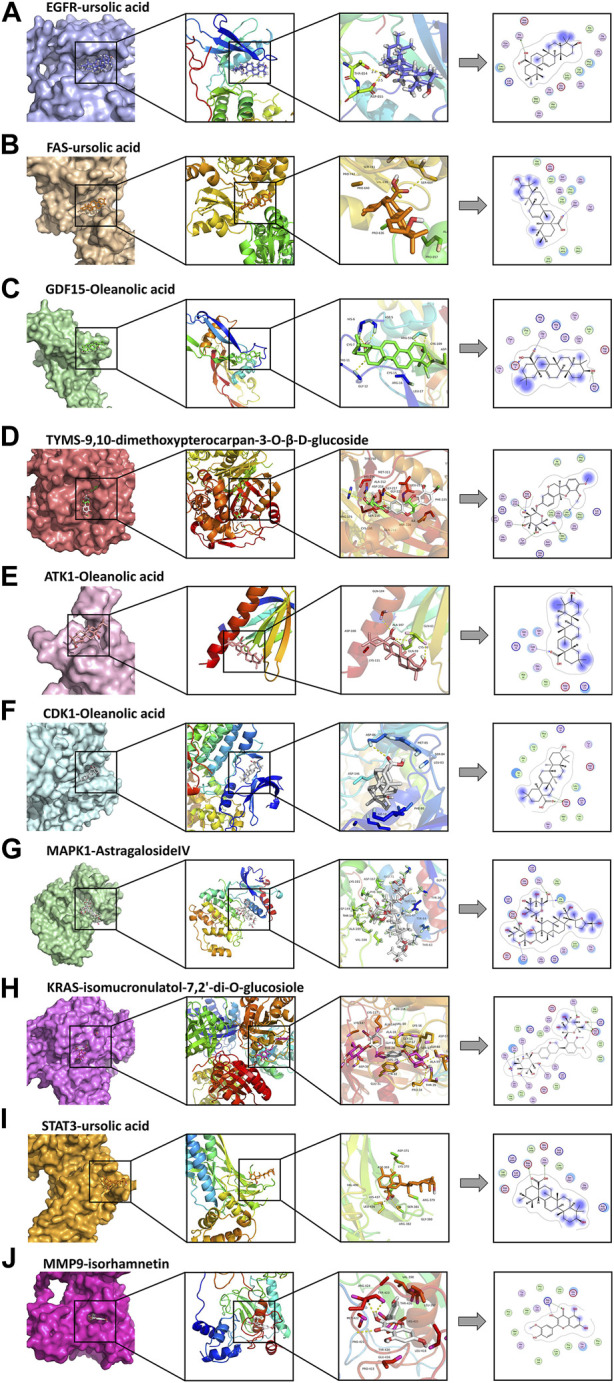
Docking complexes with the lowest binding energy: **(A)** EGFR-ursolic acid; **(B)** FAS-ursolic acid; **(C)** GDF15-Oleanolic acid; **(D)** TYMS-9,10-dimethoxypterocarpan-3-O-β-D-glucoside; **(E)** ATK1-Oleanolic acid; **(F)** CDK1-Oleanolic acid; **(G)** MAPK1-AstragalosideIV; **(H)** KRAS-isomucronulatol-7,2’-di-O-glucosiole; **(I)** STAT3-ursolic acid; **(J)** MMP9-isorhamnetin.

**FIGURE 7:**
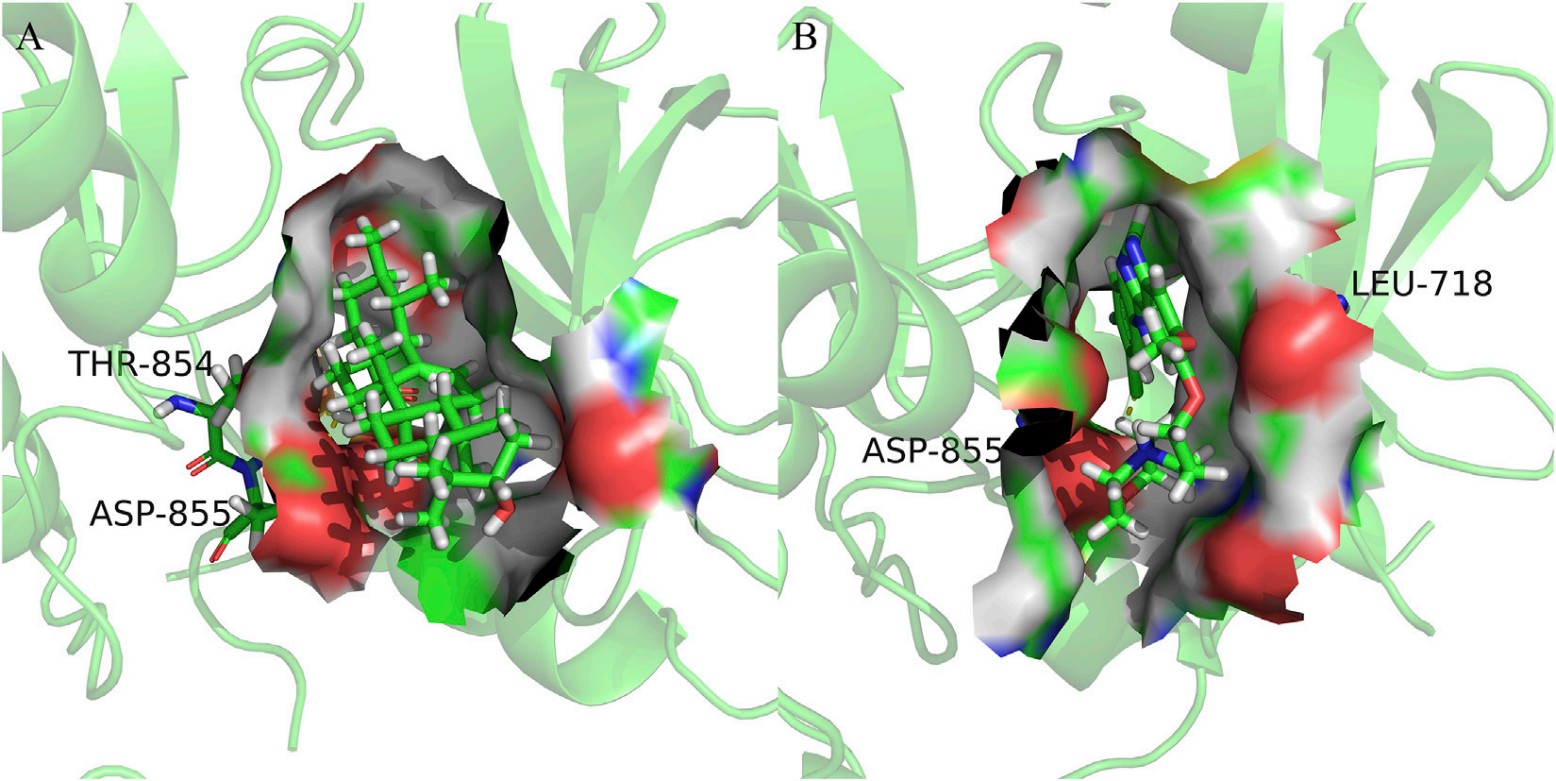
Cartoon representation of EGFR in complex with: **(A)** Ursolic acid; **(B)** Gefitinib. The binding site is shown as surface representation with the ligands shown as sticks.

The molecular docking results are illustrated in Figure 5 (the unit of measurement are kcal/mol). The redder the color, the lower binding energy and stronger affinity of the ligand-protein complex. The bluer the color, the higher the binding energy and weaker affinity of the ligand-protein complex. It is generally accepted that a compound with a binding energy less than −5.6 kcal/mol to the receptor protein indicates a strong binding (Hsin et al., 2016). The molecular docking results showed that the binding energy of most of the screened active compounds to core proteins was lower than −5.6 kcal/mol, with the binding energy of some active compounds to important targets being even less than that of Gefitinib. So, we assume that the active compounds of AR-SH can effectively treat LUAD *via* multiple targets. The complexes with lower binding energy and better conformation in each group of docking results, which were shown in Figure 5, were selected for detailed demonstration to investigate the stability of the binding, as shown in Figure 6; Figure 7 The box co-ordinates and grid size information of the protein-ligand binding sites were shown in Supplementary Table S2 **.**

We observed that UA and Gefitinib enter the same EGFR binding cavity. UA interacted with Thr-854 and Asp855 *via* hydrogen bonding and forms hydrophobic contacts with Ala722, Phe723, Val726, Ala743, Leu792, Met793 and Leu844. The interaction of UA with these residues may be the reason for its action on the target EGFR. Gefitinib forms hydrogen bonds with Asp855, as well as two π-H bonds with Leu718 and interacts with Val726, Ala743, Leu792, Met793, Leu844 and Met1002 through hydrophobic bonds.

### 3.6 MD simulation

EGFR-ursolic acid (UA), MAPK1-AstragalosideIV (ASIV) and KRAS-Isomucronulatol 7,2′-di-O-glucoside (IDOG), which are the complexes with favorable conformations and research value in the docking results, were selected for further MD simulations. MD simulations can provide a digital environmental condition like those of human cells for us, involving temperature, pressure, solvents and ions, to investigate the effects of temperature and environmental conditions on the binding process. Therefore, data obtained from MD simulations can offer valuable insights into the mechanism, dynamics, and nature of ligand-protein interactions (Wang et al., 2001).

For the information of the equilibrium time of each simulated protein-ligand complex during the MD simulation, the Root Mean Square Deviation (RMSD) of the protein backbone was calculated. RMSD is a valuable parameter for estimating changes or variations in molecular conformation, whose plots are commonly used to assess the time it takes for a system to reach structural equilibrium and to estimate the duration of the run. During the period of dramatic change in the initial structural conditions, a sudden increase of the RMSD values of the simulated complexes including the reference is expected, because the protein is rigid and would return to its dynamic motion when it is solventized in the water box in the crystal structure.

**FIGURE 8:**
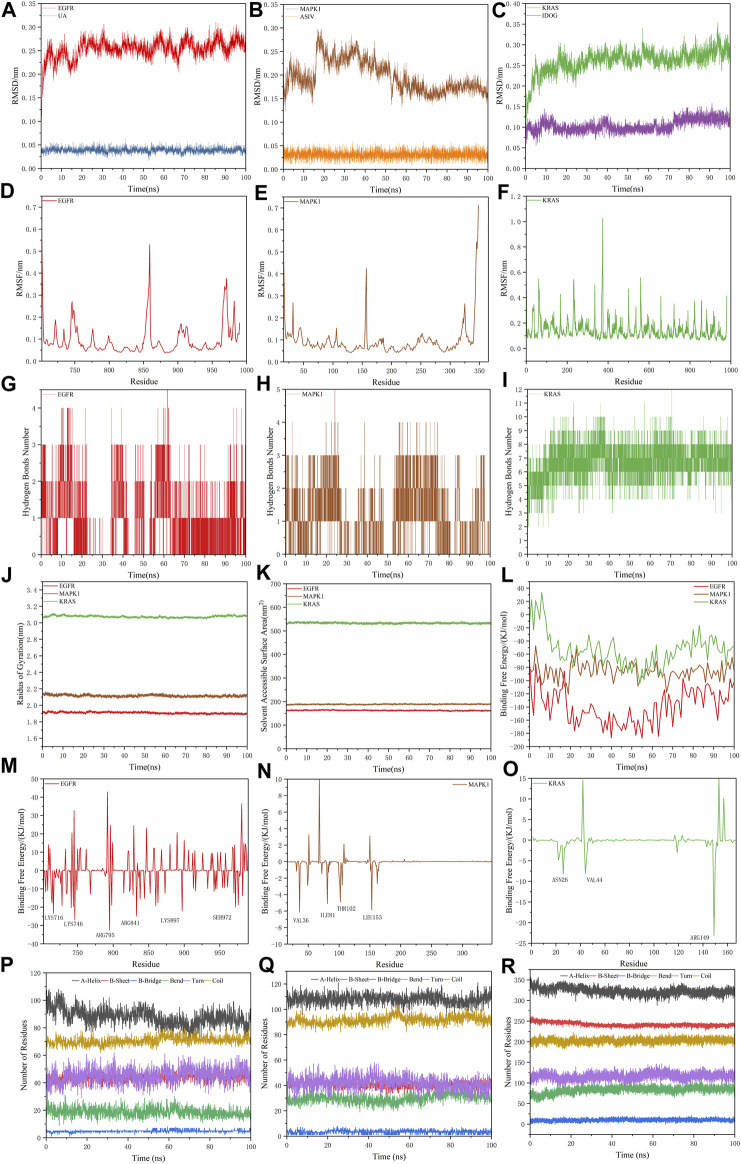
Molecular dynamics simulations. **(A–C)** The RMSD plot of EGFR-UA, MAPK1-ASIV and KRAS-IDOG. **(D–F)** The RMSF plot of EGFR-UA, MAPK1-ASIV and KRAS-IDOG. **(G–I)** The hydrogen bond numbers of EGFR-UA, MAPK1-ASIV and KRAS-IDOG. **(J,K)** Rg and SASA plots of EGFR-UA, MAPK1-ASIV and KRAS-IDOG. **(L)** Binding Free Energy plots of EGFR-UA, MAPK1-ASIV and KRAS-IDOG. **(M–O)** Binding energy contribution plots of amino acid residues of EGFR-UA, MAPK1-ASIV and KRAS-IDOG. **(P–R)** The secondary structure analysis plot of EGFR-UA, MAPK1-ASIV and KRAS-IDOG.

As shown in Figure 8A–C, the horizontal coordinates represent the time, while the vertical coordinates represent the specific values of RMSD. Sharp fluctuations of the RMSD of the three receptor-ligand complexes were witnessed in the initial stage. As the simulation proceeds, the RMSD of the three complexes tends to be smooth and stable after 20 ns. EGFR and UA were stable near 0.25 nm and 0.04 nm, MAPK1 and ASIV were stable near 0.17 nm and 0.03 nm after 50 ns, and KRAS and IDGO were stable near 0.25 nm and 0.1 nm. This phenomenon suggests that the three complexes were relatively stable in stimulated conditions (Martínez 2015). It is necessary to note that the higher the RMSD value, the more unstable the complexes (Zhao et al., 2015). Therefore, EGFR-UA exhibited greater stability.

Root Mean Square Fluctuation (RMSF) is used to examine areas with high levels of volatility, where a higher RMSF value indicates a less stable protein-ligand complex. As shown in Figures 8D–F, KRAS and the tail of MAPK1 exhibit high RMSF values, which may due to the presence of a large number of tightly coiled structures (e.g. *a*-helix and *ß*-sheet). In addition, the lower RMSF value can be caused by the loss of the corresponding structures in the complex.

Hydrogen bonding facilitates the binding capability between proteins and ligands, and the number of hydrogen bonding can reflect the induced binding affinity (Dichiara et al., 2020). As shown in Figures 8G–I, KRAS forms 6 hydrogen bonds with the ligand on average, MAPK1 forms an average of 3 hydrogen bonds with the ligand, and EGFR creates 2 hydrogen bonds on average with ligands, all of which contribute to the stable binding of the complexes.

The radius of gyration (Rg) is directly associated with the tertiary structure and overall conformational state that has been utilized to determine whether a structure has a stable, compact and folded conformation. The Larger Rg value, the more flexible proteins, and the more unstable the complexes of ligand-protein. In contrast, lower Rg values indicate densely and tightly packed protein structures (Islam et al., 2021; Dey et al., 2022). As shown in Figure 8J, EGFR and MAPK1 exhibit low Rg values, from which we thought that they are stable.

The solvent accessible surface area (SASA) can be used to describe the effective interaction between ligand complexes and receptors (Geierhaas et al., 2007), which represents the interconnection between the water molecules and the surface of the complex submerged in water molecules. SASA is based on the ratio of the total area to energy. Compounds with high SASA values form unstable protein-ligand complexes due to their easy access to solvent, while complexes with low SASA values are considered to be stable (Patel et al., 2021). Through the hydrophobic interactions in non-polar amino acids, the SASA value of the complex can be maximally reduced (Shivanika et al., 2022). In Figure 8K, EGFR and MAPK1 possess low SASA values, indicating their better stability.

Molecular mechanics Poisson-Boltzmann surface area (MM/PBSA) is an effective and reliable method for calculating the free binding energy of small inhibitors to their protein targets (Wang et al., 2017). The free binding energies of the three complexes and their changes within 100 ns of simulation are shown in Figure 8L. The average free binding energy of KRAS is −53.08 kJ/mol, the average free binding energy of MAPK1 is −85.81 kJ/mol, and the average free binding energy of EGFR is −139.21 kJ/mol. From Table 3 we could propose the complex of EGFR-UA with the best binding energy, of which the E_vdW_ and E_ele_ were both lower than that of MAPK1-ASIV. Although the E_vdW_ and E_ele_ of KRAS-IDOG were the lowest, the highest E_PB_ hindered the binding of receptor and ligand.

**TABLE 3:** Binding free energies of complexes in kJ/mol.

Complexes	G_bind_ (±SEM)	E_MM_ (±SEM)	E_vdW_ (±SEM)	E_ele_ (±SEM)	E_PB_ (±SEM)	E_SA_ (±SEM)
EGFR-UA	-139.21 ± 2.596	-212.0 ± 3.003	-167.8 ± 1.153	-44.15 ± 2.801	99.70 ± 2.732	-26.94 ± 0.1091
MAPK1-ASIV	-85.81 ± 1.284	-164.2 ± 1.078	-145.2 ± 0.8670	-18.97 ± 0.6718	97.60 ± 1.176	-19.23 ± 0.05296
KRAS-IDOG	-53.08 ± 2.546	-368.6 ± 3.087	-247.2 ± 2.245	-121.3 ± 1.926	348.3 ± 2.051	-32.83 ± 0.08525

The contribution of protein residues to free binding energy was calculated. As shown in Figures 8M–O, in the 100 ns simulation, the EGFR complex has more amino acid residues that can provide binding energy compared to the KRAS and MAPK1 complexes, indicating higher binding of ligands and receptors of the EGFR complex. Residues contributing to the free binding energy of the EGFR complex were LYS716, LYS746, ARG795, ARG841, LYS897 and SER972. Residues contributing to the binding energy of the MAPK1 complex were VAL36, ILE81, THR102 and LEU153. ASN26 VAL44 and ARG149 made significant energy contributions to the KRAS complex. These amino acid residues contributing to the free binding energy play a pivotal role in the interaction with the ligand and are the active sites for binding, which is consistent with the molecular docking results.

The secondary structure analyses of 100 ns simulated trajectory were shown in Figures 8P–R. We identified that the number of secondary structures of the three complexes including *a*-Helix, *ß*-Sheet, *ß*-Bridge, Bend, Turn and Coil is kept relatively steady, and the fluctuations get smaller as the simulation proceeds, indicating that the complexes are relatively stable.

100 ns MD simulations analysis revealed that all three complexes were stable during the simulation. Based on the RMSD, RMSF, RG, SASA, hydrogen bonding number and free binding energy, the EGFR complex emerged as the most stable complex, followed by MAPK1, which might be because of Van der Waals forces and electrostatic potential energy. KRAS complex was slightly less stable may be attributed to its more flexible protein and denser convoluted structures.

**FIGURE 9:**
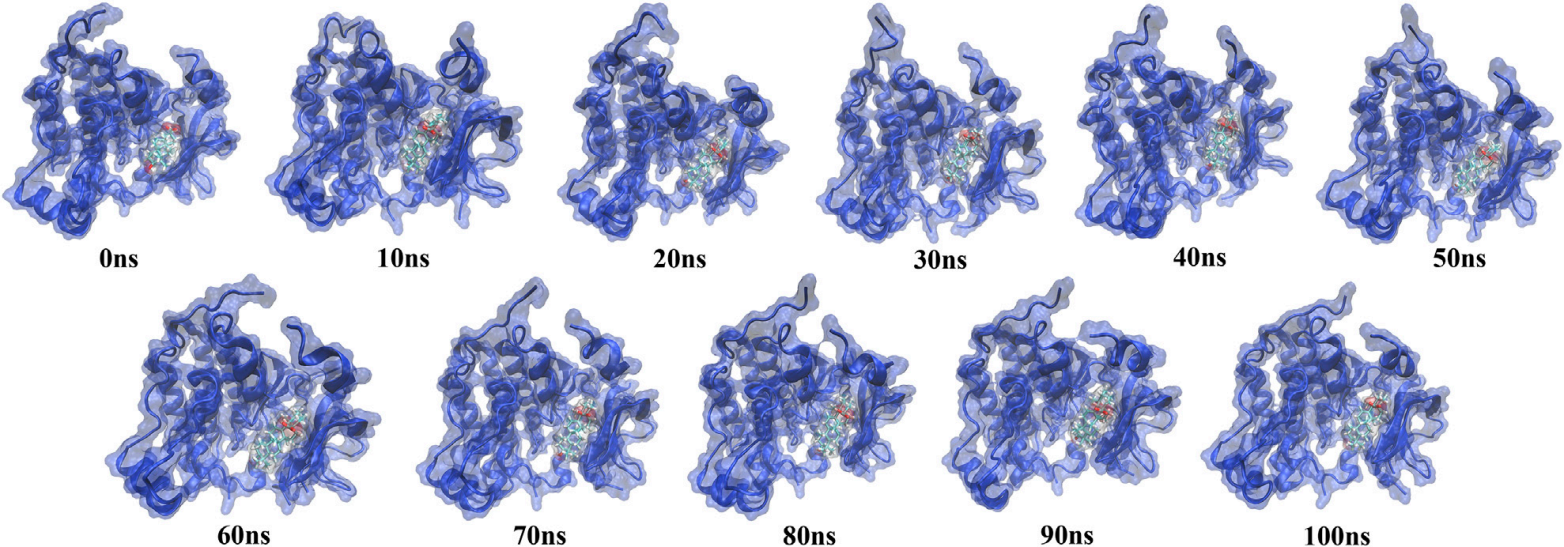
Snapshots of molecular dynamics simulations taken at 10-ns intervals that point to the movement of UA inside the binding site of EGFR.

The simulation trajectory of the EGFR-UA complex with the best stability was chosen for visualization. As displayed in Figure 9, UA can interact flexibly and gradually stabilize in the docking pocket of EGFR, with the snapshots of complexes obtained every 10 ns. As the simulation proceeded, the stability of the complex system did not change, and the ligand and protein were in a relatively static state of motion.

**FIGURE 10:**
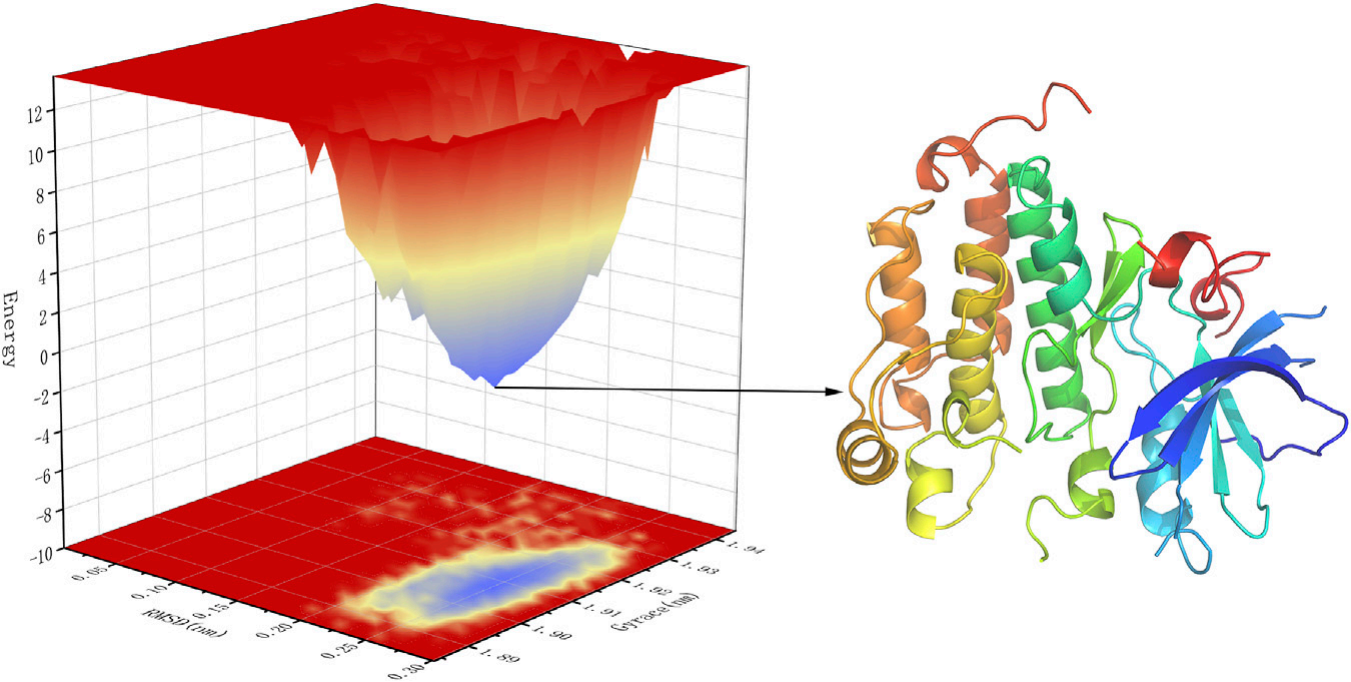
3D representation of binding free energy landscape as a function of RMSD and Gyrate. Energy distribution is shown by the coloring pattern: Blue defines the conformational space with minimum energy (stable state) while red defines a conformational space with maximum energy (unstable state). Transient local energy states are defined by intermediate color patterns.

Free energy landscape (FEL) diagram was drawn to study the relationship between structural transitions or conformational changes of proteins and free binding energy through appropriate conformational sampling procedures. RMSD and Gyrate were selected to construct 3D landscape maps to detect and explore their steady-state structures. As we can see in Figure 10, the FEL plot of EGFR-UA has a minimum in a single lowest energy well, and the free energy values are below 0 kJ/mol, indicating that the system has good stability.

## 4 Disscussion

Targets chemotherapy remains to play a leading role in the treatment for the majority of patients with advanced-stage LUAD, and EGFR-TKI is the first line drug for lung cancer patients harboring an EGFR mutation in routine clinical practice (Ciuleanu et al., 2012). EGFR-TKI has been confirmed to significantly prolong disease free survival (DFS) but not overall survival (OS) of patients compared to conventional chemotherapeutic agents (Wu et al., 2022). Nowadays, Chinese herbal medicines have been demonstrated by several studies to increase therapeutic efficiency and reduce the adverse effects of chemotherapy drugs (Tseng et al., 2016; Yang et al., 2019a; Zhang et al., 2020; Wei et al., 2022).

Herb pair of AR and SH were found to be most frequently used in the treatment of lung cancer in clinical (CHEN.H.F et al., 2022). AR, one of the most commonly used tonic herbs in clinical practice, can strengthen the spleen, and enhance the body, where modern pharmacological studies have shown that AR has a wide range of effects including hepatoprotective, diuretic, hypotensive, and immunomodulatory functions (Bedir et al., 2000). AR may inhibit the progression and metastasis of LUAD by regulating immune system such as modulating macrophage polarization (Xu et al., 2018). SH possesses heat-clearing and detoxifying properties, with several biological activities, such as neuroprotection (Kim et al., 2001) and antitumor activity (Lee et al., 2011). But there is no systematic study on the bioactive ingredients of AR-SH and the underlying mechanism of AR-SH compounds in the treatment of LUAD by now. Therefore, a network pharmacology strategy and molecular docking approach as well as molecular dynamics simulations were adopted to identify the potential targets and elucidate mechanisms of action of AR-SH in the treatment of LUAD.

A total of 29 active compounds were acquired from TCMSP using ADME parameters, and literature, with 422 targets obtained. 1381 LUAD-related targets were collected from GeneCards, OMIM and CTD databases. There are 127 common targets of AR-SH and LUAD. Among the bioactive compounds, UA is a natural pentacyclic triterpenoid with anticancer activity against a variety of cancers *in vitro* and *in vivo* (Shanmugam et al., 2011; Yang et al., 2016; Yang et al., 2019b). Wang et al. (2020) proved that UA can suppress the proliferation of various lung cancer cells, including human NSCLC cells H460, H1975, A549, H1299 and H520. Yang et al. (2019b) found that UA can inhibit the expression of CT45A2, and suppress the proliferation and motility of tumor cells while promoting apoptosis in NSCLC carrying the EGFR T790M mutation with this mutation being the main cause of drug resistance to EGFR. ASIV is a naturally occurring tetracyclic triterpene saponin that has been shown to be free of any significant hepatotoxic or nephrotoxic effects. Studies have shown that ASIV enhances the Bax/Bcl-2 ratio and induces intrinsic apoptosis in a variety of cancer cells, including cells of colorectal, breast, lung, vulvar squamous cell carcinoma, and hepatocellular carcinoma (Jia et al., 2019; Sun et al., 2019; Zhao et al., 2019; Zheng et al., 2019; Cui et al., 2020). Li et al. (2017a) suggested that ASIV can inhibit glioma progression by interfering with the MAPK/ERK signaling pathway, which is consistent with the high and stable MAPK1-ASIV binding ability in the present study, making ASIV a promising anti-cancer candidate. IDOG showed a strong binding affinity with KARS, indicating it may be a potentially active compound against cancer. Further studies should be conducted to investigate the anti-tumor effects of IDOG. In addition to the tumor suppressive function, we found that UA and ASIV can also co-regulate immune function. UA could reduce Th1 cytokine expression (IL-2, IL-6, IL-12, IFN-γ and TNF-α) and induce Th2 (IL4, IL5) cytokine expression (Raphael and Kuttan 2003; Ahmad et al., 2006). ASIV was thought to act as an immune adjuvant (Hong et al., 2011) to enhance cellular immune function by activating the NF-κB/MAPK signaling pathway (Li et al., 2017b). The cooperative anticancer activity of UA and ASIV, and the modulation of the immune system demonstrated the synergistic effect of the AR-SH drug pair.

GO enrichment analysis showed that the biological processes involved in AR-SH treatment of LUAD mainly include protein phosphorylation, negative regulation of apoptotic process, response to xenobiotic stimulus, peptide-tyrosine phosphorylation and response to drugs. KEGG pathway analysis associated with AR-SH against LUAD includes pathways of a variety of cancers, such as non-small cell lung cancer, prostate cancer, pancreatic cancer, and colorectal cancer. Cancer-related cellular alterations include endocrine resistance, EGFR tyrosine kinase inhibitor resistance, central carbon metabolism, and proteoglycans in cancer. PI3K-Akt, HIF-1 and other signaling pathways were also engaged. The emergence of drug resistance remains a major issue for EGFR-TKIs treatment of lung cancer. PI3K-Akt, an important signaling pathway present in normal human cells, is involved in a variety of physiological and pathological processes and plays a central regulatory role in cell growth and proliferation. Furthermore, PI3K-Akt pathway can also affect the development of NSCLC by inducing apoptosis, inhibiting cell proliferation, invasion and migration, and regulating tumor angiogenesis (Wang et al., 2019; Chen et al., 2020; Hu et al., 2020). HIF-1 is a key transcriptional activator that mediates the adaptive response of the organism to hypoxia, which regulates gene expression through changes in intracellular oxygen concentration and exerts an influential role in tumor cell hypoxia adaptation, energy metabolism, tumor angiogenesis, and invasion and metastasis, with its expression of HIF-1 closely related to invasive metastasis of lung cancer (Hua et al., 2020). During rapid tumor cell multiplication in patients with non-small cell lung cancer, tumor cells are in a relatively hypoxic state, making HIF-1α more likely to be activated and stay in a highly expressed stage.

Based on the reference “Inference Score” and “References” scores of each target in the CTD database and the hot spots of LUAD-related research, 10 targets were selected and considered as core targets of AR-SH for LUAD treatment, including EGFR, FAS, GDF15, TYMS, AKT1, CDK1, MAPK1, KRAS, STAT3 and MMP9. Survival analysis of core targets revealed a significant correlation between the expression of FAS (*p* = 0.046), GDF15 (*p* = 0.023), TYMS (*p* < 0.001), CDK1 (*p* < 0.001), MAPK1 (*p* = 0.027) and KRAS (*p* = 0.028) and prognosis of LUAD. However, it is undeniable that EGFR is a determinant driving lung adenocarcinoma growth and treatment response *in vivo* (Foggetti et al., 2021). EGFR and KRAS are the two most frequently mutated oncogenic driver genes (Rodenhuis et al., 1987; Lynch et al., 2004; Paez et al., 2004) that occur in the presence of multiple identified tumor suppressor gene alterations (Cancer Genome Atlas Research Network, 2014; Politi and Herbst 2015; Campbell et al., 2016; Skoulidis and Heymach 2019). EGFR, a receptor-type tyrosine kinase, is overexpressed and/or mutated in LUAD and controls tumor growth through signaling regulation. The expression of EGFR is closely associated with neo-angiogenesis, tumor invasion and metastasis (Cancer Genome Atlas Research Network, 2014), whose mutations are a major causative factor for LUAD in East Asian countries (accounting for approximately 60% of LUAD) (Dong et al., 2018). Remarkable advances have been made in the treatment of advanced NSCLC with molecularly targeted EGFR-TKIs, yet patients are highly susceptible to drug resistance (Tan et al., 2017; Dong et al., 2018). Mutation of KRAS was first initiated in lung cancer in the 1980s (Santos et al., 1984), which is a gene that is hard to target. Mutations in the KRAS gene directly trigger the EGFR-Ras-Raf-MAPK pathway in the EGF signaling pathway, followed by activation and overexpression of MAPK1 to further promote tumor cell migration and invasion, increase cell viability and participate in epithelial mesenchymal transition, allowing the rapid progress of LUAD and rendering targeted drugs against the EGFR upstream pathway ineffective (Lee et al., 2014). There exist a close relationship between mutations of KRAS and MAPK1 and the resistance of NSCLC to EGFR-TKI targeted drugs such as Gefitinib and Erlotinib, which can cause sustained activation of the EGFR signaling pathway and accelerate tumor cell proliferation (Zer et al., 2016).

What’s more, as one of the most commonly used tonic herbs, AR is not negligible for its modulating effect on the immune system. We found that the core targets EGFR, MAPK1 and KRAS are closely related to the regulation of immune system function. In the EGFR-positive genetic state, tumors exhibit a relatively immunosuppressive microenvironment, as evidenced by a decrease in CD8^+^ T cells and an increase in regulatory T cells (Treg) (Xiao et al., 2023). Of note, KRAS-mutant tumors showed a marked immune activation status in LUAD, exemplified by an elevated abundance of CD8+T cells, Cytotoxic T Lymphocyte cells (CTL), and Follicular helper T cells (Tfh), and reduced immunosuppressed M2-macrophage. In primary lung cancer, a retrospective study found KRAS-mutant tumors had a significantly higher PD-L1 expression, high CD8+T cells infiltration and higher TMB than EGFR-mutant tumors (Liu et al., 2020). Therefore, the high response rate of KRAS-positive tumors to immunotherapy may be related to the activated immune microenvironment (Lee et al., 2018). Zfp831, as a downstream molecule of MAPK1, directly binds to the Tfh cells signature gene Bcl6 and thus promotes Tfh cells differentiation (Wan et al., 2021). Consequently, we suggest that AR can modulate the immune system by acting on the core targets EGFR, MAPK1 and KRAS, thus improving the tumor immune microenvironment.

Molecular docking results showed that the binding affinities of the screened active compounds to the core targets ranged from −4.9 to −11.2 kcal/mol, with most of the ingredients exhibiting binding energies less than −5.6 kcal/mol. The binding energy of UA and oleanolic acid of SH to EGFR was even less than that of Gefitinib, while ASIV and IDOG of AR showed stronger binding to MAPK1 and KARS, respectively. It is drug pair formed from AR and SH that the effect could be reached, therefore, the synergistic effects of AR-SH on the targets may be responsible for the treatment of LUAD. Three docked complexes including UA-EGFR, ASIV-MAPK1 and IDOG-KRAS exhibited favorable docking conformations and low binding energies, and molecular dynamics simulations further suggested stability of the binding of docked complexes, with hydrogen bonding being the most critical factor for their stable binding. It was found that KRAS protein functions as a molecular switch, as it activates and regulates the downstream MAPK pathway in response to upstream EGFR, which amplifies the signaling efficiency of the MAPK pathway in KRAS mutations, ultimately controlling tumor cell proliferation and metastasis, and thus promoting tumor growth (Ponsioen et al., 2021). In this study, we found that the active ingredients of AR-SH can stably bind to EGFR, MAPK1 and KRAS to trigger or suppress their protein functions, thus contributing to the treatment of LUAD.

Although molecular dynamics simulation can be used to describe the motion of ligand-protein complex in one system, it lacks the ability to simultaneously show the interactions between the compound and other proteins which are unavoidable in human body. So, many unknown variables that cannot be controlled are stand in the way, which may have an impact accuracy of result. Despite these limitations, the molecular level analysis in this study provides a reference and guidance for further exploration of the mechanism of AR-SH for LUAD treatment. What’s more, we also found that the main active compounds of AR-SH were not acquired from databases but from literature supplementation, so we suggest that we should not rely on the database alone for active ingredient mining.

## 5 Conclusion

In conclusion, we suggested that the herb pair of AR-SH can act on targets like EGFR, MAPK1 and KRAS by UA, ASIV and IDOG, to play a vital role in the treatment and the enhancement of prognosis of LUAD.

## Data Availability

The datasets presented in this study can be found in online repositories. The names of the repository/repositories and accession number(s) can be found in the article/Supplementary Material.

## References

[B1] Ahmad S. F. Khan B. Bani S. Suri K. A. Satti N. K. Qazi G. N. (2006). Amelioration of adjuvant-induced arthritis by ursolic acid through altered Th1/Th2 cytokine production. Pharmacol. Res. 53 (3), 233–240. 10.1016/j.phrs.2005.11.005 16406805

[B2] Astragalus (2003). Astragalus membranaceus, Monograph. Altern. Med. Rev. 8 (1), 72–77. 12611564

[B3] Bamodu O. A. Kuo K. T. Wang C. H. Huang W. C. Wu A. T. H. Tsai J. T. (2019). Astragalus polysaccharides (PG2) enhances the M1 polarization of macrophages, functional maturation of dendritic cells, and T cell-mediated anticancer immune responses in patients with lung cancer. Nutrients 11 (10), 2264. 10.3390/nu11102264 31547048 PMC6836209

[B4] Bedir E. Pugh N. Calis I. Pasco D. S. Khan I. A. (2000). Immunostimulatory effects of cycloartane-type triterpene glycosides from astragalus species. Biol. Pharm. Bull. 23 (7), 834–837. 10.1248/bpb.23.834 10919362

[B5] Bray F. Ferlay J. Soerjomataram I. Siegel R. L. Torre L. A. Jemal A. (2018). Global cancer statistics 2018: GLOBOCAN estimates of incidence and mortality worldwide for 36 cancers in 185 countries. CA A Cancer J. Clin. 68 (6), 394–424. 10.3322/caac.21492 30207593

[B6] Bussi G. Donadio D. Parrinello M. (2007). Canonical sampling through velocity rescaling. J. Chem. Phys. 126 (1), 014101. 10.1063/1.2408420 17212484

[B7] Campbell J. D. Alexandrov A. Kim J. Wala J. Berger A. H. Pedamallu C. S. (2016). Distinct patterns of somatic genome alterations in lung adenocarcinomas and squamous cell carcinomas. Nat. Genet. 48 (6), 607–616. 10.1038/ng.3564 27158780 PMC4884143

[B8] Cancer Genome Atlas Research Network (2014). Comprehensive molecular profiling of lung adenocarcinoma. Nature 511 (7511), 543–550. 10.1038/nature13385 25079552 PMC4231481

[B9] Chen H. F. Lei X. D. Dang H. F. Li.Y. H. Wang H. Xia X. J. (2022). The study of the law of drug use for the treatment of lung cancer by Chinese medicine. Gansu Med. J. 41 (02), 134–136. 10.15975/j.cnki.gsyy.2022.02.023

[B10] Chen M. Zhu L. L. Su J. L. Li G. L. Wang J. Zhang Y. N. (2020). Prucalopride inhibits lung cancer cell proliferation, invasion, and migration through blocking of the PI3K/AKT/mTor signaling pathway. Hum. Exp. Toxicol. 39 (2), 173–181. 10.1177/0960327119883409 31640407

[B11] Chen T. Yang P. Jia Y. (2021). Molecular mechanisms of astragaloside-IV in cancer therapy (Review). Int. J. Mol. Med. 47 (3), 4846. 10.3892/ijmm.2021.4846 PMC7834967 33448320

[B12] Ciuleanu T. Stelmakh L. Cicenas S. Miliauskas S. Grigorescu A. C. Hillenbach C. (2012). Efficacy and safety of erlotinib versus chemotherapy in second-line treatment of patients with advanced, non-small-cell lung cancer with poor prognosis (TITAN): A randomised multicentre, open-label, phase 3 study. Lancet Oncol. 13 (3), 300–308. 10.1016/s1470-2045(11)70385-0 22277837

[B13] Cui X. Jiang X. Wei C. Xing Y. Tong G. (2020). Astragaloside IV suppresses development of hepatocellular carcinoma by regulating miR-150-5p/β-catenin axis. Environ. Toxicol. Pharmacol. 78, 103397. 10.1016/j.etap.2020.103397 32417721

[B14] De Vivo M. Masetti M. Bottegoni G. Cavalli A. (2016). Role of molecular dynamics and related methods in drug discovery. J. Med. Chem. 59 (9), 4035–4061. 10.1021/acs.jmedchem.5b01684 26807648

[B15] Dey D. Hossain R. Biswas P. Paul P. Islam M. A. Ema T. I. (2022). Amentoflavone derivatives significantly act towards the main protease (3CL(PRO)/M(PRO)) of SARS-CoV-2: In silico admet profiling, molecular docking, molecular dynamics simulation, network pharmacology. Mol. Divers 2022, 1–15. 10.1007/s11030-022-10459-9 PMC9153225 35639226

[B16] Dichiara M. Amata B. Turnaturi R. Marrazzo A. Amata E. (2020). Tuning properties for blood-brain barrier permeation: A statistics-based analysis. ACS Chem. Neurosci. 11 (1), 34–44. 10.1021/acschemneuro.9b00541 31793759

[B17] Dong J. K. Lei H. M. Liang Q. Tang Y. B. Zhou Y. Wang Y. (2018). Overcoming erlotinib resistance in EGFR mutation-positive lung adenocarcinomas through repression of phosphoglycerate dehydrogenase. Theranostics 8 (7), 1808–1823. 10.7150/thno.23177 29556358 PMC5858502

[B18] Duflos A. Kruczynski A. Barret J. M. (2002). Novel aspects of natural and modified vinca alkaloids. Curr. Med. Chem. Anticancer Agents 2 (1), 55–70. 10.2174/1568011023354452 12678751

[B19] Efferth T. Li P. C. Konkimalla V. S. Kaina B. (2007). From traditional Chinese medicine to rational cancer therapy. Trends Mol. Med. 13 (8), 353–361. 10.1016/j.molmed.2007.07.001 17644431

[B20] Eguchi T. Kodera Y. Nakanishi H. Yokoyama H. Ohashi N. Ito Y. (2008). The effect of chemotherapy against micrometastases and isolated tumor cells in lymph nodes: An *in vivo* study. Vivo 22 (6), 707–712. 19180995

[B21] Foggetti G. Li C. Cai H. Hellyer J. A. Lin W. Y. Ayeni D. (2021). Genetic determinants of EGFR-driven lung cancer growth and therapeutic response *in vivo* . Cancer Discov. 11 (7), 1736–1753. 10.1158/2159-8290.Cd-20-1385 33707235 PMC8530463

[B22] Fu J. Wang Z. Huang L. Zheng S. Wang D. Chen S. (2014). Review of the botanical characteristics, phytochemistry, and pharmacology of Astragalus membranaceus (Huangqi). Phytother. Res. 28 (9), 1275–1283. 10.1002/ptr.5188 25087616

[B23] Geierhaas C. D. Nickson A. A. Lindorff-Larsen K. Clarke J. Vendruscolo M. (2007). BPPred: A web-based computational tool for predicting biophysical parameters of proteins. Protein Sci. 16 (1), 125–134. 10.1110/ps.062383807 17123959 PMC2222837

[B24] Hert J. Willett P. Wilton D. J. Acklin P. Azzaoui K. Jacoby E. (2004). Comparison of topological descriptors for similarity-based virtual screening using multiple bioactive reference structures. Org. Biomol. Chem. 2 (22), 3256–3266. 10.1039/b409865j 15534703

[B25] Homeyer N. Gohlke H. (2012). Free energy calculations by the molecular mechanics Poisson-Boltzmann surface area method. Mol. Inf. 31 (2), 114–122. 10.1002/minf.201100135 27476956

[B26] Hong F. Xiao W. Ragupathi G. Lau C. B. Leung P. C. Yeung K. S. (2011). The known immunologically active components of Astragalus account for only a small proportion of the immunological adjuvant activity when combined with conjugate vaccines. Planta Med. 77 (8), 817–824. 10.1055/s-0030-1250574 21128203 PMC3711077

[B27] Hsin K. Y. Matsuoka Y. Asai Y. Kamiyoshi K. Watanabe T. Kawaoka Y. (2016). systemsDock: a web server for network pharmacology-based prediction and analysis. Nucleic Acids Res. 44 (1), W507–W513. 10.1093/nar/gkw335 27131384 PMC4987901

[B28] Hu T. Shen H. Huang H. Yang Z. Zhou Y. Zhao G. (2020). Cholesterol-lowering drug pitavastatin targets lung cancer and angiogenesis via suppressing prenylation-dependent Ras/Raf/MEK and PI3K/Akt/mTOR signaling. Anticancer Drugs 31 (4), 377–384. 10.1097/cad.0000000000000885 32011362

[B29] Hua Q. Mi B. Xu F. Wen J. Zhao L. Liu J. (2020). Hypoxia-induced lncRNA-AC020978 promotes proliferation and glycolytic metabolism of non-small cell lung cancer by regulating PKM2/HIF-1α axis. Theranostics 10 (11), 4762–4778. 10.7150/thno.43839 32308748 PMC7163453

[B30] Islam R. Parves M. R. Paul A. S. Uddin N. Rahman M. S. Mamun A. A. (2021). A molecular modeling approach to identify effective antiviral phytochemicals against the main protease of SARS-CoV-2. J. Biomol. Struct. Dyn. 39 (9), 3213–3224. 10.1080/07391102.2020.1761883 32340562 PMC7232885

[B31] Jia L. Lv D. Zhang S. Wang Z. Zhou B. (2019). Astragaloside IV inhibits the progression of non-small cell lung cancer through the akt/GSK-3β/β-catenin pathway. Oncol. Res. 27 (4), 503–508. 10.3727/096504018x15344989701565 30131090 PMC7848426

[B32] Kim Y. Park E. J. Kim J. Kim Y. Kim S. R. Kim Y. Y. (2001). Neuroprotective constituents from Hedyotis diffusa. J. Nat. Prod. 64 (1), 75–78. 10.1021/np000327d 11170670

[B33] Kollman P. A. Massova I. Reyes C. Kuhn B. Huo S. Chong L. (2000). Calculating structures and free energies of complex molecules: Combining molecular mechanics and continuum models. Acc. Chem. Res. 33 (12), 889–897. 10.1021/ar000033j 11123888

[B34] Kumari R. Kumar R. Lynn A. (2014). g_mmpbsa--a GROMACS tool for high-throughput MM-PBSA calculations. J. Chem. Inf. Model 54 (7), 1951–1962. 10.1021/ci500020m 24850022

[B35] Lee B. Lee B. Han G. Kwon M. J. Han J. Choi Y. L. (2014). KRAS mutation detection in non-small cell lung cancer using a peptide nucleic acid-mediated polymerase chain reaction clamping method and comparative validation with next-generation sequencing. Korean J. Pathol. 48 (2), 100–107. 10.4132/KoreanJPathol.2014.48.2.100 24868222 PMC4026800

[B36] Lee C. K. Man J. Lord S. Cooper W. Links M. Gebski V. (2018). Clinical and molecular characteristics associated with survival among patients treated with checkpoint inhibitors for advanced non-small cell lung carcinoma: A systematic review and meta-analysis. JAMA Oncol. 4 (2), 210–216. 10.1001/jamaoncol.2017.4427 29270615 PMC5838598

[B37] Lee H. Z. Bau D. T. Kuo C. L. Tsai R. Y. Chen Y. C. Chang Y. H. (2011). Clarification of the phenotypic characteristics and anti-tumor activity of Hedyotis diffusa. Am. J. Chin. Med. 39 (1), 201–213. 10.1142/s0192415x11008750 21213409

[B38] Li B. Meng T. Hao N. Tao H. Zou S. Li M. (2017b). Immune regulation mechanism of Astragaloside IV on RAW264.7 cells through activating the NF-κB/MAPK signaling pathway. Int. Immunopharmacol. 49, 38–49. 10.1016/j.intimp.2017.05.017 28550733

[B39] Li B. Wang F. Liu N. Shen W. Huang T. (2017a). Astragaloside IV inhibits progression of glioma via blocking MAPK/ERK signaling pathway. Biochem. Biophys. Res. Commun. 491 (1), 98–103. 10.1016/j.bbrc.2017.07.052 28709870

[B40] Li Y. L. Zhang J. Min D. Hongyan Z. Lin N. Li Q. S. (2016). Anticancer effects of 1,3-dihydroxy-2-methylanthraquinone and the ethyl acetate fraction of Hedyotis diffusa willd against HepG2 carcinoma cells mediated via apoptosis. PLoS One 11 (4), e0151502. 10.1371/journal.pone.0151502 27064569 PMC4827846

[B41] Liang Y. N. Hou B. L. Wu K. N. Wu D. Y. Pei G. Wang. Z. (2022). Study on chemical components from Hedyotis diffusa Willd and their anti-tumour activity. Nat. Prod. Res. Dev. 34 (08), 1281–1288+1300. 10.16333/j.1001-6880.2022.8.002

[B42] Liao Y. H. Li C. I. Lin C. C. Lin J. G. Chiang J. H. Li T. C. (2017). Traditional Chinese medicine as adjunctive therapy improves the long-term survival of lung cancer patients. J. Cancer Res. Clin. Oncol. 143 (12), 2425–2435. 10.1007/s00432-017-2491-6 28803328 PMC11819392

[B43] Lin J. Li Q. Chen H. Lin H. Lai Z. Peng J. (2015). Hedyotis diffusa Willd. extract suppresses proliferation and induces apoptosis via IL-6-inducible STAT3 pathway inactivation in human colorectal cancer cells. Oncol. Lett. 9 (4), 1962–1970. 10.3892/ol.2015.2956 25789077 PMC4356405

[B44] Lin J. Wei L. Xu W. Hong Z. Liu X. Peng J. (2011). Effect of Hedyotis diffusa willd extract on tumor angiogenesis. Mol. Med. Rep. 4 (6), 1283–1288. 10.3892/mmr.2011.577 21887465

[B45] Liu C. Zheng S. Jin R. Wang X. Wang F. Zang R. (2020). The superior efficacy of anti-PD-1/PD-L1 immunotherapy in KRAS-mutant non-small cell lung cancer that correlates with an inflammatory phenotype and increased immunogenicity. Cancer Lett. 470, 95–105. 10.1016/j.canlet.2019.10.027 31644929

[B46] Liu Z. Liu M. Liu M. Li J. (2010). Methylanthraquinone from Hedyotis diffusa WILLD induces Ca(2+)-mediated apoptosis in human breast cancer cells. Toxicol Vitro 24 (1), 142–147. 10.1016/j.tiv.2009.08.002 19686834

[B47] Lynch T. J. Bell D. W. Sordella R. Gurubhagavatula S. Okimoto R. A. Brannigan B. W. (2004). Activating mutations in the epidermal growth factor receptor underlying responsiveness of non-small-cell lung cancer to gefitinib. N. Engl. J. Med. 350 (21), 2129–2139. 10.1056/NEJMoa040938 15118073

[B48] Martínez L. (2015). Automatic identification of mobile and rigid substructures in molecular dynamics simulations and fractional structural fluctuation analysis. PLoS One 10 (3), e0119264. 10.1371/journal.pone.0119264 25816325 PMC4376797

[B49] Miller B. R. 3rd McGee T. D. Jr. Swails J. M. Homeyer N. Gohlke H. Roitberg A. E. (2012). MMPBSA.py: An efficient program for end-state free energy calculations. J. Chem. Theory Comput. 8 (9), 3314–3321. 10.1021/ct300418h 26605738

[B50] Noguchi M. Morikawa A. Kawasaki M. Matsuno Y. Yamada T. Hirohashi S. (1995). Small adenocarcinoma of the lung. Histologic characteristics and prognosis. Cancer 75 (12), 2844–2852. 10.1002/1097-0142(19950615)75:12<2844::aid-cncr2820751209>3.0.co;2-# 7773933

[B51] Paez J. G. Jänne P. A. Lee J. C. Tracy S. Greulich H. Gabriel S. (2004). EGFR mutations in lung cancer: Correlation with clinical response to gefitinib therapy. Science 304 (5676), 1497–1500. 10.1126/science.1099314 15118125

[B52] Patel C. N. Goswami D. Jaiswal D. G. Parmar R. M. Solanki H. A. Pandya H. A. (2021). Pinpointing the potential hits for hindering interaction of SARS-CoV-2 S-protein with ACE2 from the pool of antiviral phytochemicals utilizing molecular docking and molecular dynamics (MD) simulations. J. Mol. Graph Model 105, 107874. 10.1016/j.jmgm.2021.107874 33647752 PMC7897937

[B53] Politi K. Herbst R. S. (2015). Lung cancer in the era of precision medicine. Clin. Cancer Res. 21 (10), 2213–2220. 10.1158/1078-0432.Ccr-14-2748 25979927 PMC4505624

[B54] Ponsioen B. Post J. B. Buissant des Amorie J. R. Laskaris D. van Ineveld R. L. Kersten S. (2021). Quantifying single-cell ERK dynamics in colorectal cancer organoids reveals EGFR as an amplifier of oncogenic MAPK pathway signalling. Nat. Cell Biol. 23 (4), 377–390. 10.1038/s41556-021-00654-5 33795873 PMC7610573

[B55] Raphael T. J. Kuttan G. (2003). Effect of naturally occurring triterpenoids glycyrrhizic acid, ursolic acid, oleanolic acid and nomilin on the immune system. Phytomedicine 10 (6-7), 483–489. 10.1078/094471103322331421 13678231

[B56] Rodenhuis S. van de Wetering M. L. Mooi W. J. Evers S. G. van Zandwijk N. Bos J. L. (1987). Mutational activation of the K-ras oncogene. A possible pathogenetic factor in adenocarcinoma of the lung. N. Engl. J. Med. 317 (15), 929–935. 10.1056/nejm198710083171504 3041218

[B57] Rogers D. Hahn M. (2010). Extended-connectivity fingerprints. J. Chem. Inf. Model 50 (5), 742–754. 10.1021/ci100050t 20426451

[B58] Santos E. Martin-Zanca D. Reddy E. P. Pierotti M. A. Della Porta G. Barbacid M. (1984). Malignant activation of a K-ras oncogene in lung carcinoma but not in normal tissue of the same patient. Science 223 (4637), 661–664. 10.1126/science.6695174 6695174

[B59] Shanmugam M. K. Manu K. A. Ong T. H. Ramachandran L. Surana R. Bist P. (2011). Inhibition of CXCR4/CXCL12 signaling axis by ursolic acid leads to suppression of metastasis in transgenic adenocarcinoma of mouse prostate model. Int. J. Cancer 129 (7), 1552–1563. 10.1002/ijc.26120 21480220

[B60] Sher T. Dy G. K. Adjei A. A. (2008). Small cell lung cancer. Mayo Clin. Proc. 83 (3), 355–367. 10.4065/83.3.355 18316005

[B61] Shivanika C. Deepak Kumar S. Venkataraghavan Ragunathan R. Sumitha A. Brindha Devi P. (2022). Molecular docking, validation, dynamics simulations, and pharmacokinetic prediction of natural compounds against the SARS-CoV-2 main-protease. J. Biomol. Struct. Dyn. 40 (2), 585–611. 10.1080/07391102.2020.1815584 32897178 PMC7573242

[B62] Siegel R. L. Miller K. D. Jemal A. (2019). Cancer statistics, 2019. CA Cancer J. Clin. 69 (1), 7–34. 10.3322/caac.21551 30620402

[B63] Skoulidis F. Heymach J. V. (2019). Co-occurring genomic alterations in non-small-cell lung cancer biology and therapy. Nat. Rev. Cancer 19 (9), 495–509. 10.1038/s41568-019-0179-8 31406302 PMC7043073

[B64] Sun P. Liu Y. Wang Q. Zhang B. (2019). Astragaloside IV inhibits human colorectal cancer cell growth. Front. Biosci. (Landmark Ed. 24 (3), 597–606. 10.2741/4738 30468676

[B65] Tan C. S. Cho B. C. Soo R. A. (2017). Treatment options for EGFR mutant NSCLC with CNS involvement-Can patients BLOOM with the use of next generation EGFR TKIs? Lung Cancer 108, 29–37. 10.1016/j.lungcan.2017.02.012 28625644

[B66] Tian X. Y. Liu L. (2010). Effect and advantage of orally taking Chinese herbal medicine for treatment of lung cancer. China J. Chin. Materia Medica 35 (21), 2795–2800. 21322934

[B67] Tseng C. Y. Lin C. H. Wu L. Y. Wang J. S. Chung M. C. Chang J. F. (2016). Potential combinational anti-cancer therapy in non-small cell lung cancer with traditional Chinese medicine Sun-Bai-pi extract and cisplatin. PLoS One 11 (5), e0155469. 10.1371/journal.pone.0155469 27171432 PMC4865219

[B68] Wan S. Ni L. Zhao X. Liu X. Xu W. Jin W. (2021). Costimulation molecules differentially regulate the ERK-Zfp831 axis to shape T follicular helper cell differentiation. Immunity 54 (12), 2740–2755.e6. 10.1016/j.immuni.2021.09.018 34644536

[B69] Wang C. Greene D. Xiao L. Qi R. Luo R. (2017). Recent developments and applications of the MMPBSA method. Front. Mol. Biosci. 4, 87. 10.3389/fmolb.2017.00087 29367919 PMC5768160

[B70] Wang G. Zhu W. (2016). Molecular docking for drug discovery and development: A widely used approach but far from perfect. Future Med. Chem. 8 (14), 1707–1710. 10.4155/fmc-2016-0143 27578269

[B71] Wang J. Wolf R. M. Caldwell J. W. Kollman P. A. Case D. A. (2004). Development and testing of a general amber force field. J. Comput. Chem. 25 (9), 1157–1174. 10.1002/jcc.20035 15116359

[B72] Wang M. Yu H. Wu R. Chen Z. Y. Hu Q. Zhang Y. F. (2020). Autophagy inhibition enhances the inhibitory effects of ursolic acid on lung cancer cells. Int. J. Mol. Med. 46 (5), 1816–1826. 10.3892/ijmm.2020.4714 32901853 PMC7521584

[B73] Wang Q. Liu Z. Du K. Liang M. Zhu X. Yu Z. (2019). Babaodan inhibits cell growth by inducing autophagy through the PI3K/AKT/mTOR pathway and enhances antitumor effects of cisplatin in NSCLC cells. Am. J. Transl. Res. 11 (8), 5272–5283. 31497240 PMC6731395

[B74] Wang W. Donini O. Reyes C. M. Kollman P. A. (2001). Biomolecular simulations: Recent developments in force fields, simulations of enzyme catalysis, protein-ligand, protein-protein, and protein-nucleic acid noncovalent interactions. Annu. Rev. Biophys. Biomol. Struct. 30, 211–243. 10.1146/annurev.biophys.30.1.211 11340059

[B75] Wei J. Liu Z. He J. Liu Q. Lu Y. He S. (2022). Traditional Chinese medicine reverses cancer multidrug resistance and its mechanism. Clin. Transl. Oncol. 24 (3), 471–482. 10.1007/s12094-021-02716-4 34643878

[B76] Wu Y. L. John T. Grohe C. Majem M. Goldman J. W. Kim S. W. (2022). Postoperative chemotherapy use and outcomes from ADAURA: Osimertinib as adjuvant therapy for resected EGFR-mutated NSCLC. J. Thorac. Oncol. 17 (3), 423–433. 10.1016/j.jtho.2021.10.014 34740861

[B77] Xiao G. Li L. Tanzhu G. Liu Z. Gao X. Wan X. (2023). Heterogeneity of tumor immune microenvironment of EGFR/ALK-positive tumors versus EGFR/ALK-negative tumors in resected brain metastases from lung adenocarcinoma. J. Immunother. Cancer 11 (3). 10.1136/jitc-2022-006243 PMC9990629 36868569

[B78] Xu F. Cui W. Q. Wei Y. Cui J. Qiu J. Hu L. L. (2018). Astragaloside IV inhibits lung cancer progression and metastasis by modulating macrophage polarization through AMPK signaling. J. Exp. Clin. Cancer Res. 37 (1), 207. 10.1186/s13046-018-0878-0 30157903 PMC6116548

[B79] Yang K. Chen Y. Zhou J. Ma L. Shan Y. Cheng X. (2019a). Ursolic acid promotes apoptosis and mediates transcriptional suppression of CT45A2 gene expression in non-small-cell lung carcinoma harbouring EGFR T790M mutations. Br. J. Pharmacol. 176 (24), 4609–4624. 10.1111/bph.14793 31322286 PMC6965687

[B80] Yang K. Li R. Y. Yang X. Y. Cui Q. F. Wang F. Y. Lin G. Q. (2019b). Co-Administration of shexiang baoxin pill and chemotherapy drugs potentiated cancer therapy by vascular-promoting strategy. Front. Pharmacol. 10, 565. 10.3389/fphar.2019.00565 31178734 PMC6543272

[B81] Yang L. J. Tang Q. Wu J. Chen Y. Zheng F. Dai Z. (2016). Inter-regulation of IGFBP1 and FOXO3a unveils novel mechanism in ursolic acid-inhibited growth of hepatocellular carcinoma cells. J. Exp. Clin. Cancer Res. 35, 59. 10.1186/s13046-016-0330-2 27036874 PMC4815122

[B82] Yue D. Xu S. Wang Q. Li X. Shen Y. Zhao H. (2018). Erlotinib versus vinorelbine plus cisplatin as adjuvant therapy in Chinese patients with stage IIIA EGFR mutation-positive non-small-cell lung cancer (EVAN): A randomised, open-label, phase 2 trial. Lancet Respir. Med. 6 (11), 863–873. 10.1016/s2213-2600(18)30277-7 30150014

[B83] Zappa C. Mousa S. A. (2016). Non-small cell lung cancer: Current treatment and future advances. Transl. Lung Cancer Res. 5 (3), 288–300. 10.21037/tlcr.2016.06.07 27413711 PMC4931124

[B84] Zer A. Ding K. Lee S. M. Goss G. D. Seymour L. Ellis P. M. (2016). Pooled analysis of the prognostic and predictive value of KRAS mutation status and mutation subtype in patients with non-small cell lung cancer treated with epidermal growth factor receptor tyrosine kinase inhibitors. J. Thorac. Oncol. 11 (3), 312–323. 10.1016/j.jtho.2015.11.010 26749487

[B85] Zhang C. Cai T. Zeng X. Cai D. Chen Y. Huang X. (2018). Astragaloside IV reverses MNNG-induced precancerous lesions of gastric carcinoma in rats: Regulation on glycolysis through miRNA-34a/LDHA pathway. Phytother. Res. 32 (7), 1364–1372. 10.1002/ptr.6070 29577459

[B86] Zhang Y. Lou Y. Wang J. Yu C. Shen W. (2020). Research status and molecular mechanism of the traditional Chinese medicine and antitumor therapy combined strategy based on tumor microenvironment. Front. Immunol. 11, 609705. 10.3389/fimmu.2020.609705 33552068 PMC7859437

[B87] Zhao Y. Wang L. Wang Y. Dong S. Yang S. Guan Y. (2019). Astragaloside IV inhibits cell proliferation in vulvar squamous cell carcinoma through the TGF-β/Smad signaling pathway. Dermatol Ther. 32 (4), e12802. 10.1111/dth.12802 30536730

[B88] Zhao Y. Zeng C. Massiah M. A. (2015). Molecular dynamics simulation reveals insights into the mechanism of unfolding by the A130T/V mutations within the MID1 zinc-binding Bbox1 domain. PLoS One 10 (4), e0124377. 10.1371/journal.pone.0124377 25874572 PMC4395243

[B89] Zheng Y. Dai Y. Liu W. Wang N. Cai Y. Wang S. (2019). Astragaloside IV enhances taxol chemosensitivity of breast cancer via caveolin-1-targeting oxidant damage. J. Cell Physiol. 234 (4), 4277–4290. 10.1002/jcp.27196 30146689

[B90] Zhong W. Z. Wang Q. Mao W. M. Xu S. T. Wu L. Wei Y. C. (2021). Gefitinib versus vinorelbine plus cisplatin as adjuvant treatment for stage II-IIIA (N1-N2) EGFR-mutant NSCLC: Final overall survival analysis of CTONG1104 phase III trial. J. Clin. Oncol. 39 (7), 713–722. 10.1200/jco.20.01820 33332190 PMC8078324

